# Exploring the Bioavailability of Red Grape Skin Extract Polyphenols: A Caco-2 Cell Model Study

**DOI:** 10.3390/foods14132253

**Published:** 2025-06-25

**Authors:** Edurne Elejalde, Rosa María Alonso, María Carmen Villarán, Lucía Díez-Gutiérrez, María Chávarri, Iratxe López-de-Armentia

**Affiliations:** 1 TECNALIA, Basque Research and Technology Alliance (BRTA), Parque Tecnológico de Álava, 01510 Miñano, Álava, Spain; edurne.elejalde@tecnalia.com (E.E.); mcarmen.villaran@tecnalia.com (M.C.V.); ld27@sanger.ac.uk (L.D.-G.); maria.chavarri@tecnalia.com (M.C.); iratxe.lopezdearmentia@tecnalia.com (I.L.-d.-A.); 2 FARMARTEM Group, Analytical Chemistry Department, Faculty of Science and Technology, University of the Basque Country (UPV/EHU), 48940 Leioa, Biscay, Spain

**Keywords:** grape skin, polyphenols, in vitro bioavailability, intestinal permeability, Caco-2 cells

## Abstract

Grapes are a rich source of polyphenols with a positive impact on human health. Polyphenols need to be bioavailable to exert any beneficial effect. However, there is limited knowledge on the bioavailability of polyphenols in grape extracts. The intestinal permeability of nine polyphenols of a red grape skin extract (GSE) was investigated using the Caco-2 cell model that simulates the human intestinal epithelium: three anthocyanins (delphinidin-3-*O*-glucoside, petunidin-3-*O*-glucoside and malvidin-3-*O*-glucoside), three flavonols (quercetin-3-glucoside, kaempferol-3-galactoside and kaempferol-3-glucoside), two hydroxybenzoic acids (gallic acid and syringic acid) and one hydroxycinnamic acid (caftaric acid). Two concentrations of GSE (15 mg/mL and 22 mg/mL) were used. The transport efficiency (TE) through the Caco-2 monolayer was studied. Among anthocyanins, only malvidin-3-*O*-glucoside was detected at the basolateral side, which represents the bloodstream, with a TE of 1.08 ± 0.01%. Flavonols resulted in a variety of results depending on the GSE concentration. Among flavonols, kaempferol-3-glucoside showed the highest TE of 130 ± 3%. Gallic acid showed the highest TE among the investigated polyphenols with 188 ± 3%. This study provides data on the intestinal transport of red grape skin extract polyphenols that can be used to explore the underlying mechanisms of the intestinal absorption and the bioactivity of natural grape extracts.

## 1. Introduction

Polyphenols constitute a diverse group of secondary metabolites found in several plant sources, including fruits, vegetables, herbs and seeds, making them the most prevalent phytochemicals in the plant kingdom [1]. Polyphenols have attracted much interest due to their preventive effect on cardiovascular diseases, diabetes and cancer [2,3,4,5]. Moreover, they have also been reported to have excellent properties as food preservatives due to their antimicrobial and antioxidant activities [6], as nutraceuticals [7] and as supplements for athletes to reduce the negative effect of exercise-induced oxidative stress, accelerating the recovery of muscular function and enhancing performance [8,9]. Many other industrial applications as natural colorants for foods and for the production of cosmetics [10,11] have also been reported.

Grape is one of the most popular fruits in the world and one with the highest polyphenol content [12,13]. Due to its extensive global cultivation, the grape is considered one of the most significant fruit crops [14]. In fact, more than 80 million tons of grapes are produced worldwide each year. Among these, 74.5 million tons are used to produce wine, must, juices, table grapes and dried grapes, but 5.6 million tons are lost [15]. In the context of circular bioeconomy, the valorization of grapes to obtain grape extracts is of great interest. Consequently, a detailed analysis of grape extracts is required to assess their viability as natural sources of bioactive compounds for nutraceutical applications.

Over 1600 different molecules have been found in grapes; the polyphenols are the most relevant due to their biological activities and health promoting benefits [16]. The grape polyphenols are mainly distributed in the seeds, the skin and the pulp. Polyphenols, like flavonols and anthocyanins are mainly localized in the skin, whereas the flavan-3-ols are present both in the skin and seeds. Therefore, grape skin is an excellent source of natural bioactive compounds such as polyphenols but also fatty acids, higher alcohols, sterols and terpenes [17,18]. With this relevant polyphenolic profile, grape skin extracts are considered first-rate candidates for the development of nutraceuticals.

Polyphenols have attracted considerable interest due to their diverse range of biological effects [19], playing a crucial role in the prevention of several major chronic diseases associated with oxidative stress such as cardiovascular diseases, cancer, type II diabetes, neurodegenerative diseases or osteoporosis [20,21]. However, research on intestinal absorption rates of polyphenols extracted from grape skins is still limited [22,23,24,25].

The proportion of a compound absorbed by the intestinal wall and passing into the bloodstream is defined as bioavailability, which is influenced by several factors such as the matrix and the surrounding substances. The Caco-2 cell line is commonly used as a model for in vitro intestinal absorption assays and to evaluate the bioavailability of nutrients and drugs in the human body [26,27]. Caco-2 cells were originally isolated in 1977 from a 72-year-old colorectal cancer patient at the Sloan–Kettering Institute for Cancer Research (New York, NY, USA) [28]. These cells can create a dense and continuous monolayer growing on porous Transwell permeable support polycarbonate membranes like Transwell inserts (e.g., Transwell). When cultured in an appropriate medium, Caco-2 cells undergo spontaneous enterocytic differentiation and polarization, making them a suitable model system for the small intestinal epithelium. Furthermore, they exhibit morphological and biochemical characteristics similar to those of enterocytes (e.g., polarity, tight junctions, specific transport systems and enzymes) [29].

The Caco-2 cell model of absorption has been tested to be a good alternative for in vivo studies. It has good reproducibility [30,31] and has emerged as one of the standard in vitro tools to predict in vivo human intestinal absorption for phenolic compounds [32]. In fact, the European Medicines Agency (EMA) and Food and Drug Administration (FDA) have recognized the Caco-2 cell line as a reliable in vitro model for predicting the bioavailability of drugs in the pharmaceutical industry [33,34,35]. Despite existing research on grape pomace polyphenols bioavailability [36,37,38,39], the information regarding grape skin polyphenols bioavailability is still limited [40]. This study fills this knowledge gap by studying the intestinal transport of a polyphenol-rich red grape skin extract with potential to be considered a health-promoting extract and a valuable source of bioactive compounds.

Therefore, the main purpose of this study was to evaluate the intestinal permeability of the main polyphenols with health beneficial effects present in a red grape skin extract in a Caco-2 cell model.

## 2. Materials and Methods

### 2.1. Chemicals and Reagents

Dulbecco’s Modified Eagle’s Medium (DMEM) of GIBCO was supplied by Fisher Scientific (Göteborg, Sweden). Fetal bovine serum (FBS), Hank’s Balanced Salt Solution (HBSS) and phosphate-buffered saline were (PBS) also obtained through Merck Life Science (Madrid, Spain). The Caco-2 human colon adenocarcinoma (Homo sapiens) cell line, source ATCC, catalogue number ATCC-HTB-37, was supplied by LGC Standards (Barcelona, Spain). Non-essential amino acid (NEAA), sodium bicarbonate 7.5%, 4-(2-hydroxyethyl)-1-piperazineethanesulfonic acid (HEPES), Lonza™ Trypsin-Versene™-Trypsin-EDTA cell culture reagent and 3-[4,5-dimethylthiazol-2-yl]-2,5 diphenyl tetrazolium bromide (MTT) were supplied by Fisher Scientific (Göteborg, Sweden). Dimethyl sulfoxide (DMSO), acetonitrile HPLC gradient grade and trifluoroacetic acid buffer substance HPLC grade were obtained through Scharlab (Barcelona, Spain).

The reference standards for HPLC malvidin-3-*O*-glucoside (M3G) with ≥95% purity, delphinidin-3-*O*-glucoside (D3G) with ≥95% purity, petunidin-3-*O*-glucoside (Pet3G) with ≥96% purity, quercetin-3-glucoside (Q3G) with ≥98% purity, kaempferol-3-galactoside (K3Gal) with ≥98% purity and kaempferol-3-glucoside (K3G) with ≥99% purity were supplied by Extrasynthese (Genai, France). The rest of reference standards for HPLC analysis gallic acid (GA) with ≥98% purity, syringic acid (SA) with ≥95% purity and caftaric acid (CA) with ≥97% purity were purchased from Merck Life Science (Madrid, Spain). The ultrapure water used was Milli-Q (Merck Millipore, Darmstadt, Germany).

### 2.2. Grape Samples

Experiments were conducted with the red grape variety Tempranillo (*Vitis vinifera*), considered one the most important red grape varieties worldwide for winemaking. It is also one of the most widely planted grape varieties in Spain [41,42].

Grape samples were provided by Bodegas Baigorri, S.A.U. winery under Rioja Qualified Designation of Origin. Grape bunches were collected in the vineyard in October 2022 at their maturity point for winemaking. They were processed as previously shown [43].

First, the grape samples were transported directly to the laboratory, followed by immediate freezing in liquid nitrogen. Afterward, the grapes were manually separated into skin, pulp and seeds. The skins were freeze-dried (Lyobeta 6PL; Telstar, Terrassa, Barcelona, Spain) by applying a constant vacuum chamber pressure of 0.030 mbar and a condenser temperature of −45 °C, for 48 h. Finally, the dried skins were stored in vacuum bags in the dark at −20 °C until extraction.

### 2.3. Preparation of Polyphenolic Grape Skin Extract

To obtain the polyphenolic grape skin extracts, ultrasound-assisted extraction was carried out in a Bioblock Scientific Vibra Cell VCX 750 sonicator (Sonde standard 13 mm, Fisher Scientific, Madrid, Spain) at a frequency of 20 KHz. 2 g of the freeze-dried grape skin was placed in a beaker with 100 mL of 50% ethanol (*v*/*v*) and ultrasound assisted extraction was applied for 20 min. The conditions for the extraction procedure were established according to the literature [44,45,46]. After treatment, the liquid extract was centrifuged (5810R Eppendorf; Merck Life Science, Madrid, Spain) at 2.150× *g* at 4 °C for 5 min and filtered using Whatman 0.45 µm nylon filter paper. Ethanol was eliminated by a rotatory evaporator (R-205 Büchi, Fisher Scientific, Madrid, Spain). The aqueous extract was freeze-dried (Lyobeta 6PL; Telstar, Barcelona, Spain) to obtain the dried grape skin polyphenolic extract (GSE) which was stored in the dark at −20 °C. For subsequent research, a stock solution of GSE was prepared at a concentration of 75 mg/mL in H_2_O.

### 2.4. HPLC Analysis of Polyphenols

Briefly, HPLC analysis of the polyphenols of the GSE and the samples in the bioavailability study was performed using an Agilent 1260 Infinity II LC System (Agilent Technologies, Santa Clara, CA, USA) equipped with a thermostatically controlled column (T = 25 °C) compartment and a diode array detector. A reversed phase C18 column (Luna 5 μm C18(2) 100 Å, LC Column 250 × 4.6 mm, Phenomenex) was used to separate the polyphenols at a flow rate of 0.7 mL/min with an injection volume of 60 µL. The detection was carried out at 520 nm for anthocyanins (M3G, D3G and Pet3G), at 328 nm for CA, at 356 nm for flavonols (Q3G, K3Gal and K3G) and at 280 nm for GA and SA. The mobile phases were (A) acetonitrile 100% and (B) water 0.1% trifluoroacetic acid (*v*/*v*). The gradient was programmed as follows: 10% A 0 min, 10–80% A (0–20 min), 80–100% A (20–25 min), 100% A (25–30 min); 10% A (30–35 min) and 10% A (35–40 min). Every polyphenol was quantified using the corresponding reference standard calibration curve. Each of the quantified phenolic compound concentration was expressed as mg/mL

### 2.5. Cell Culture

#### 2.5.1. Caco-2 Cell Growth Conditions

Human Caco-2 cells were grown in DMEM supplemented with 20% (*v*/*v*) FBS, 1% (*v*/*v*) non-essential amino acids, 2% (*w*/*v*) sodium bicarbonate and 0.63% (*w*/*v*) of 4-(2-hydroxyethyl)-1-piperazineethanesulfonic acid. 1 × 10^5^ cells per ml were cultured in 75 cm^2^ flasks at 37 °C in a humidified incubator containing 5% CO_2_. During cell growth, the medium was renewed every 3 days.

#### 2.5.2. Cytotoxicity Evaluation

The cytotoxic effect of GSE was evaluated using 96-well plates (Corning, Fisher Scientific, Madrid, Spain) to screen the best concentration range to carry out the transepithelial electrical resistance (TEER) assay, before the bioavailability study. The effect of the grape skin extract on cell viability was measured by the 3-[4,5-dimethylthiazol-2-yl]-2,5 diphenyl tetrazolium bromide (MTT) assay [47,48]. Therefore, Caco-2 cells were collected from the 75 cm^2^ flasks and 5 × 10^3^ cells were supplied per well. After 24 h of incubation at 37 °C in a humidified atmosphere containing 5% CO_2_, dilutions ranging between 1 to 18 mg/mL of the GSE stock solution were prepared in DMEM. Caco-2 cells in DMEM without GSE were used as control. 100 µL of each GSE solution were added to each well (four wells in total) of each plate (three plates in total). After 24 h of incubation, the grape skin extract was removed, 100 µL of fresh media and 30 µL of MTT with a concentration of 5 mg/mL were added. After 4 h of incubation, MTT was discarded and 100 µL of DMSO were pipetted onto each well to dissolve the formazan crystals produced by the metabolism of MTT by Caco-2 cells. The solubilized formazan crystals produced a purple colour solution whose absorbance was measured at 570 nm using a microplate photometer (Multiskan™ FC; Fisher Scientific, Madrid, Spain).

There is a linear relationship between cell viability and absorbance. The results are expressed as a percentage of viability, compared to untreated control cells (without the addition of grape skin extract). Each experiment was repeated three times.

#### 2.5.3. TEER Assay and Bioavailability Study

For the transport experiment Caco-2 cells were cultured at a density of 4 × 10^4^ cells/cm^2^ in Transwell inserts (12 mm i.d., 1.12 cm^2^ growth area, 0.4 µm mean pore size polycarbonate membranes, Corning Inc., Milwaukee, WI, USA) placed in 12-well plates. Caco-2 cells were grown in DMEM in an incubator at 37 °C in a humidified atmosphere containing 5% CO_2_.

Every 3 days, the media was changed, and TEER was measured (Millipore) to ensure the continuity of the cell monolayer and to exclude any effects due to insert damage. TEER values were recorded in culture medium at 37 °C and expressed as Ω cm^2^, according to Equation (1):

TEER = (Ω cell monolayer − Ω insert cell free) × insert area(1)

where, Ω cell monolayer is the TEER measurement of electrical resistance across the Caco-2 cell monolayer; Ω insert cell free is the TEER value of the empty insert and filter area is the effective area of the insert in cm^2^.

Bioavailability studies were performed in triplicate after 21 days of cell growth. Only inserts with stable TEER values higher than ≥300 Ω cm^2^ were considered acceptable to perform the bioavailability study [49]. For that purpose, DMEM media was removed, the monolayer was washed with PBS and HBSS was supplied to the cells.

Likewise, the GSE stock solution was diluted in HBSS medium to prepare GSE solutions S1 and S2. GSE solutions S1 and S2 had concentrations of 15 mg/mL and 22 mg/mL, respectively. These concentrations were established considering previous studies with grape pomace extracts that concluded that intestinal concentrations of 1–2% (*w*/*v*) show potential bioactivities and are safe to be used as a food ingredient [50].

Before the bioavailability studies, TEER was measured before and after 180 min of exposure to the GSE solutions (S1 and S2) to confirm the Caco-2 monolayer integrity.

The bioavailability studies of GSE solutions (S1 and S2) were carried out by sampling 100 µL of the solution in contact with the Caco-2 cell monolayer at 0, 75, 90, 115 and 180 min, both in apical (AP) and basolateral (BL) side, and followed by adding an equal volume of fresh culture medium. The apical side represents the intestinal lumen and the basolateral side represents the bloodstream [26]. Each sample (100 µL) was immediately acidified with 1M HCl to a final concentration of 0.007 M and subsequently frozen at −20 °C until analysis by HPLC (according to the method described in Section 2.5). Each experiment was carried out on 3 individual wells. The transport efficiency (TE) percentage was calculated according to Equation (2):

Transport efficiency (%) = (C/C_0_) × 100 (2)

where, C is the polyphenol concentration at the acceptor side over time and C_0_ is the initial concentration at the donor side. TE values were calculated for the nine polyphenols identified in the grape skin extract.

The apparent permeability coefficient (P_app_) of the polyphenols in GSE was calculated for apical to basolateral side (AB) and basolateral to apical side (BA) flux studies according to Equation (3):

P_app_ (cm/s) = (dQ/dt) × (V_R_/AC_0_) (3)

where, dQ/dt is the permeability rate which means the steady-state linear rate of change of concentration (mg/L) per second (s) at the acceptor side; A is the surface area of the cell monolayer (1.12 cm^2^). C_0_ is the initial concentration at the donor side (mg/L) at t = 0 s and V_R_ is the volume of the solution of the acceptor side (mL).

The efflux ratio (ER) [51] of the polyphenol was assessed by calculating the Equation (4):

ER = Papp (BA)/Papp (AB)(4)

where, P_app_ (BA) is the apparent permeability coefficient of the polyphenol from basolateral to apical side and P_app_ (AB) is the apparent permeability coefficient from apical to basolateral side. ER is a measure of the relative contributions of active and passive transport to bi-direction flux across the cell monolayer [52].

### 2.6. Statistical Analysis

Results are reported as mean ± standard deviation (SD) of at least three independent batches for each sample. Data were analyzed using one-way ANOVA and Fisher’s Least Significant Difference (LSD) tests to estimate the differences between sample values, with a statistical significance level of *p* < 0.05. Statgraphics Centurion 18 SP XVII software was used for statistical analysis.

## 3. Results and Discussion

### 3.1. Polyphenolic Composition of Grape Skin Extract

**Figure 1:**
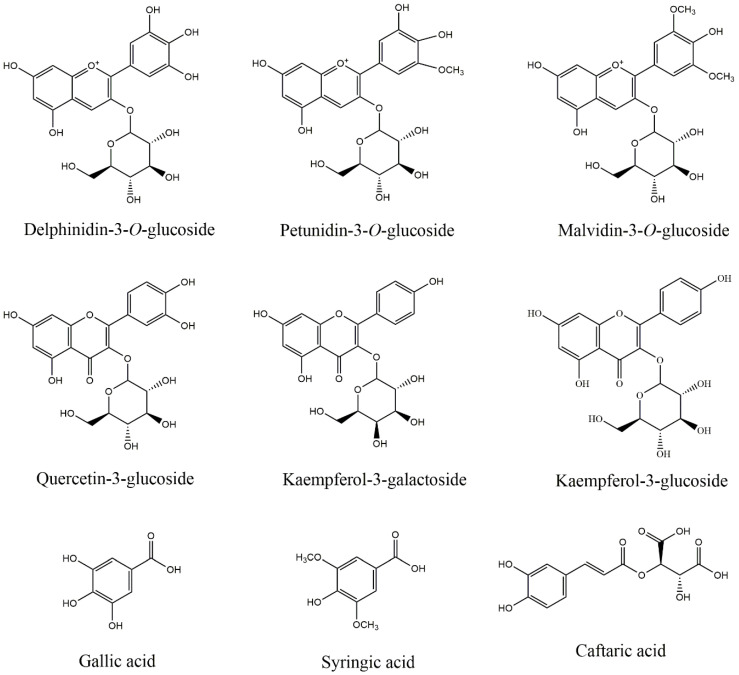
Chemical structures of the polyphenols present in the grape skin extract.

Nine polyphenols (Figure 1) were quantified in the grape skin extract (GSE) derived from the Tempranillo red grape variety. These polyphenols represent the major phenolic families in grape skin, such as anthocyanins, flavonols and phenolic acids, highlighted due to their biological activities [17,53,54].

The polyphenolic composition in Table 1 is referred to dried GSE.

The composition of the grape skin extract was consistent with previous works [10,55,56]. The polyphenolic profile is characteristic of the cultivar and highly influenced by viticulture and environmental factors such as sunlight, temperature, latitude, soil type and water and nutritional status [57,58]. The anthocyanin M3G was the major individual polyphenol present in the extract followed by the flavonol K3G.

### 3.2. Cytotoxicity of Grape Skin Extract

**Figure 2:**
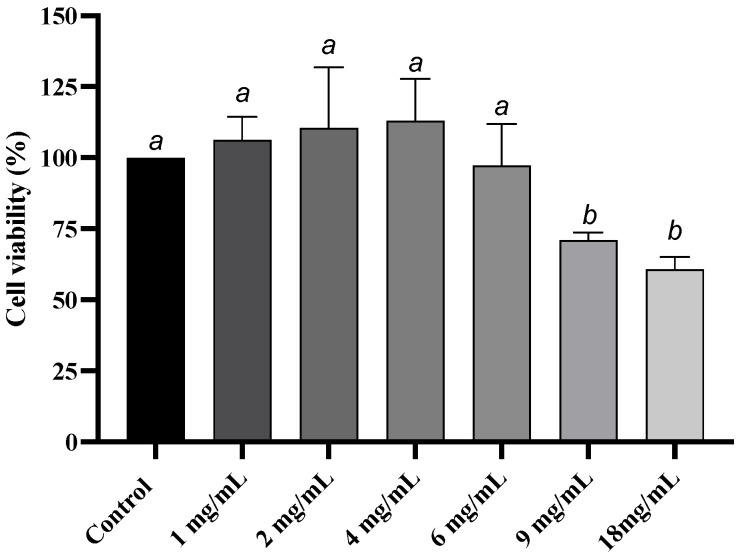
Viability of Caco-2 cells by MTT assay at different GSE concentrations in DMEM. Caco-2 cells in DMEM with no GSE serves as control. Different letters indicate statistical differences by One-way ANOVA followed by Fisher’s Least Significant Difference (LSD) test (*p* < 0.05). Values are expressed as mean (*n* = 3) ± standard deviation (SD).

The viability of Caco-2 cells at different concentrations of the GSE was calculated to determine the cytotoxic effect of GSE after 24 h of treatment. Figure 2 indicates that Caco-2 cell viability remained unaffected by GSE concentrations ranging from 1 to 6 mg/mL.

A concentration higher than 9 mg/mL reduced the viability significantly (*p* < 0.05) of after 24 h. The coefficients of variation for 1 to 6 mg/mL ranged from 7.7% to 14.9%. However, the coefficients of variation for 9 mg/mL and 18 mg/mL were 3.7% and 7.0%, respectively.

Overall, the cytotoxicity of the GSE was significantly lower than the grape pomace extracts tested in previous studies [19,59]. Unlike grape pomace, the grape extract used in this study is derived from an unfermented red grape variety. Consequently, the grape extract exhibits a distinct polyphenolic profile, which may contribute to its lower cytotoxicity.

### 3.3. Transport of Grape Skin Extract Polyphenols in the Human Colorectal Adenocarcinoma Cells Caco-2 Monolayer

Two different GSE concentrations, 15 mg/mL (S1) and 22 mg/mL (S2), were tested for 180 min. Since S1 and S2 concentrations were higher than 9 mg/mL, obtained in the 24 h cytotoxicity test, TEER measurements were performed to verify the integrity of the Caco-2 cell monolayer for the subsequent 180 min transport study. Table 2 summarizes the TEER values measured before the addition of S1 and S2 and 180 min after exposure.

TEER values remained stable at approximately 90% regardless of the GSE solution. Therefore, S1 and S2 concentrations were found to be suitable for the bioavailability study. They maintained the integrity of the cell layer throughout the transport experiments.

Transepithelial transport studies were carried out in the apical to basolateral (AB) and basolateral to apical (BA) directions using the Caco-2 cell monolayer model for S1 and S2 GSE solutions. For each experiment (AB and BA) both apical and basolateral sampling was done at time 0 min, 75 min, 90 min, 115 min and 180 min. The concentration of each polyphenol was determined in all samples using HPLC-DAD as mentioned in Materials and Methods in Section 2.4.

Some polyphenols were not always detected in either direction, and occasionally only in one of them.

The concentrations of the polyphenols that were not detected on the acceptor side are given below for the AB (see Table 3) and BA (see Table 4) directions and for the GSE solutions S1 (15 mg/mL) and S2 (22 mg/mL).

Moreover, Table 5 and Table 6 show the concentration of the polyphenols that were detected on the acceptor side. Table 5 shows the concentration of the polyphenols detected in the basolateral side in AB experiments for S1 (15 mg/mL) and S2 (22 mg/mL) GSE solutions. Table 6 shows the concentration of the polyphenols detected in the apical side in BA experiments, for S1 (15 mg/mL) and S2 (22 mg/mL) GSE solutions.

The results demonstrated a reduction in different manner with time and concentration for each polyphenol. In AB experiments (see Table 3) the concentration of each polyphenol decreased linearly on the donor side, regardless of the initial GSE concentration. With regards to the percentage reduction, the results revealed a greater reduction for all compounds at lower initial GSE concentrations, indicating a concentration-dependent effect. Besides, independently the initial GSE concentration, the SA reduction rate was the highest and the quickest in comparison to the flavonol K3G and both anthocyanins D3G and Pet3G. The reduction rate for SA was of 67 ± 4% (from 0.70 ± 0.03 to 0.23 ± 0.03) for the lowest initial GSE concentration and 55 ± 2% (from 1.48 ± 0.06 to 0.67 ± 0.03) for the highest. Moreover, at 115 min, the reduction for all the polyphenols was almost the maximum showing a possible saturation of the transport in the Caco-2 cell monolayer. These results are in accordance with those of authors who did not found accumulation of anthocyanins like D3G at the basolateral side [60].

In BA experiments (see Table 4) the tendency was also linear for all the polyphenols. In these experiments K3Gal was the polyphenol that showed the greatest reduction in the S1 sample, decreasing by with 81 ± 2% (from 0.26 ± 0.01 to 0.05 ± 0.01). However, in sample S2 the polyphenol with the greatest reduction was SA with 23 ± 1% (from 1.2 ± 0.1 to 0.90 ± 0.08). Furthermore, the reduction rate reached its maximum in approximately 75 min. While Q3G was not found at the acceptor side in the AB experiments (see Table 3), it was present at the acceptor side in the BA experiments (see Table 6).

Thus, the fact that only a few GSE polyphenols were able to pass across the Caco-2 cell monolayer after 180 min in both AB or BA experiments (see Table 5 and Table 6) could be attributed to the complex composition of the grape skin extract. The presence of different polyphenols, beyond other possible compounds in the extract, could lead to the saturation of specific transporters present in the cell monolayer complicating or avoiding the permeation to the receiver side. Several cell transporters have been reported to be affected by the presence of polyphenols such as SGLT1 [61,62], GLT2 [63], GLT5 [63] and P-GP [64]. Furthermore, it must be considered that Caco-2 cell could metabolize the analyzed polyphenols avoiding their detection in the acceptor side. Moreover, it must be considered that several authors [65] indicated that polyphenolic compounds show a significant capacity to bind phospholipid membranes of the Caco-2 cells which could also explain the obtained results.

Among all the polyphenols determined in the original GSE, in AB experiments four compounds were detected at the acceptor side: M3G, K3G, GA and CA (see Table 5). In BA experiments besides M3G, K3G, Ga and SA, Q3G were also detected (see Table 6).

Each polyphenol showed a differential transport efficiency at every specific time. Among the anthocyanins analyzed in GSE, only M3G showed the ability to cross the Caco-2 intestinal cell barrier, albeit only with the highest concentration of GSE extract (S2). Some authors have explained that the stability of anthocyanins appears to be improved by a fewer number of free hydroxyl groups and a greater number of methoxy groups in the B-ring of the molecule [66]. The chemical structure of the anthocyanins affects the extend of transport and the rate across the Caco-2 cell barrier [67]. Taking into account the structure of the analyzed anthocyanins, M3G is the most stable anthocyanin due to a greater number of methoxy groups in the B ring followed by Pet3G and D3G. Considering the aforementioned factors and the significantly higher concentration of M3G in the initial GSE compared to D3G (2.6 times) and Pet3G (2.8 times) (Table 1), the authors consider that these factors are likely to be main reasons for the detection of M3G in both the AB and BA experiments. In AB experiments the apical loss of M3G was associated with the enrichment in the basolateral compartment, only detected at the highest concentration of the GSE solution (S2). The reduction in concentration of M3G on the apical side after 180 min ranged from 75 ± 2% (from 6.1 ± 0.3 to 1.53 ± 0.04) at the lowest GSE concentration solution (S1) to 58 ± 3% (from 15 ± 1 to 6.0 ± 0.8) for the highest GSE concentration solution (S2). On the basolateral side, M3G was undetectable at the S1 concentration, whereas at the S2 concentration, a transport efficiency of 1.08 ± 0.01% was observed after 180 min. The M3G transport efficiency results reported in this study are lower than those observed in previous research, where transport efficiencies of 3% to 5% after 180 min [58] and 0.35% after 240 min [54] have been documented. Regarding other polyphenol sources, some authors [68] demonstrated a transepithelial transport M3G efficiency in Caco-2 cells ranging from 6.93–7.31% depending on the initial rabbiteye blueberry extract concentration after 2 h. In BA experiments the M3G transport efficiency towards the acceptor side for S1 was about 3.2 ± 0.3% and 1.73 ± 0.04% for S2, higher than in AB experiments, thereby indicating that M3G was more inclined to be transported to the AP side.

Q3G was not detected at the acceptor side in AB experiments, independently of the GSE concentration (see Table 3). However, in BA experiments, Q3G was quantified in the acceptor side (see Table 6). Other studies with Caco-2 cell model have shown that when analyzing cell lysates in experiments with undigested and digested wine samples [69], Q3G was found exclusively in the cell lysate, suggesting that 3-*O*-glucosylation of this flavonol could improve its absorption in the small intestine. This could explain that Q3G could be absorbed by the Caco-2 cell, avoiding its transport to the basolateral side.

Regarding K3G, the results showed in the AB experiment a 97 ± 2% transport efficiency for S1 and a lower transport efficiency for the most concentrated GSE solution (S2) with 60 ± 1%. However, the reduction in the apical side for both concentrations was quite similar of 33.6 ± 0.5% (from 7.7 ± 0.3 to 5.0 ± 0.2) for S1 and 28.7 ± 0.9% (from 12.7 ± 0.3 to 9.0 ± 0.2) for S2. For the BA experiments, the transport efficiency reached 120 ± 3% for S1 and 109.01 ± 0.08% for S2. These results showed a higher concentration on the acceptor side than the initial concentration in the donor side. Considering the extract and the complexity of this extract our hypothesis is that after 75 min, other polyphenols different from the ones that have been analyzed could have been metabolized by the Caco-2 cells producing more simple compounds as K3G. These results can be seen in both AB and BA experiments. Regarding flavonols, it has been described that the Caco-2 cell transporter Pgp, which is an efflux transporter, not only exports the polyphenols into the cytoplasm of the Caco-2, but it therefore pumps them out, even the generated metabolites. In fact, it is known that such an efflux pump gives protection to our body against xenobiotics present in our diet [64] which could explain the increase of K3G in the acceptor side.

For GA, the results showed a linear GA reduction in the donor side for both AB and BA experiments and a linear increase for both solutions S1 and S2 in the acceptor side (see Table 5 and Table 6). The transport efficiency in AB experiments was of 156 ± 26% for S1 and of 164 ± 23% for S2, while for the BA experiments the transport efficiency reached the value of 165 ± 6% for S1 and 188 ± 4% for S2. Several conclusions obtained in other studies could explain these results. Specifically, at the intestinal level GA might result in spontaneous chemical degradation of delphinidin glycosides considering its limited stability in cell culture medium [70]. Moreover, M3G has been proven to be completely metabolized to SA after 24 h of incubation [71] and a demethylation of this compound through microbial processes can led to GA [72]. These results could explain the increase of GA in the donor side in AB and BA experiments through the metabolization of the polyphenols present in the GSE to form polyphenol-derived metabolites by Caco-2 cells [73].

Regarding CA, this polyphenol exhibited a transport efficiency in AB experiments of 7 ± 1% for S1 and 4.4 ± 0.5% for S2. However, the apical reduction was of 59 ± 4% (from 1.04 ± 0.08 to 0.43 ± 0.07) for S1 and 44.1 ± 0.7% (1.82 ± 0.03 to 1.01 ± 0.01) for S2. The decrease of CA on the apical side exceeded by far the appearance on the basolateral side, indicating that additional factors might contribute to the metabolization of this compound by Caco-2 cells [74]. In BA experiments the transport efficiency was of 11.3 ± 0.8% for S1 and 8.7 ± 0.9% for S2. In BA experiments, the reduction was of 43.5 ± 0.4% for S1 and 22.6 ± 0.8% for S2.

Afterwards, the P_app_ coefficient based on Equation (3) was obtained. This value was only calculated for M3G, K3G, GA and CA considering that these polyphenols were the ones detected on the acceptor side for both AB and BA experiments.

Additionally, efflux ratio (ER) was measured. See Table 7.

The apparent permeability coefficients P_app_ for GSE polyphenols were at the level of 10^−4^ cm/s. These P_app_ results are higher compared to other studies with P_app_ at 10^−6^ cm/s [75], demonstrating that the polyphenols in the GSE have higher permeability through Caco-2 cell monolayer. The presence of other polyphenols in GSE assisted the transport across the Caco-2 cell monolayer which has been previously well proved in other natural extracts [76].

Furthermore, these P_app_ results are according to other authors that showed a higher transport efficiency with the lowest extract concentration [68]. This may be due to the saturation of transport proteins on the Caco-2 cell membrane. The greater GSE polyphenolic concentration, the more rapid cell membrane transporters saturation, which could cause the reduction of the transport efficiency through the Caco-2 cell. However, for GA, the apparent permeability was slightly increased with GSE concentration.

The efflux ratio for M3G (S2), and K3G and CA was higher than 1. These results demonstrated a higher permeability value (P_app_) in the basolateral to apical direction than its permeability in opposite direction, suggesting that the permeability value is dependent on direction. This observation supports the idea that the secretion rate of transport for these molecules (P_app BA_) direction was greater than the absorptive rate of transport (P_app AB_) direction. However, for GA the efflux ratio was less than 1 demonstrating a higher permeability in the AB direction. In general, efflux ratio results greater than 2 is an indicative of active efflux in which the molecule is actively pumped back into the intestinal lumen, resulting in reducing its intestinal absorption. The values equal to 1 indicate a passive diffusion and the values lower than 1 an active influx. When the efflux ratio result is between 1 and 2, the result suggests a passive diffusion transport mechanism.

## 4. Conclusions

This work represents for the first time a bioavailability study in a Caco-2 cell model of polyphenols belonging to different families in a grape skin extract.

The results provide valuable insights into the in vitro permeability of grape skin polyphenols at the intestinal level considering their potential as nutraceuticals. These findings underscore the high permeation of M3G, Q3G, K3G, GA and CA present in GSE, highlighting the effect of the matrix on the bioavailability of the polyphenols. Other polyphenols D3G, Pet3G, K3Gal and SA were not detected at the acceptor side of the Caco-2 cell monolayer.

In summary, this study has provided for the first time a comprehensive insight into the impact of a polyphenolic extract derived from a natural source, such as grape skin, on the bioavailability of polyphenols. Specifically, it has been observed that the complex polyphenol matrix influences the individual bioavailability of each polyphenol. More studies are imperative to understand the interrelationship among GSE polyphenols in the bioavailability, whether synergistic, antagonistic, or neutral effects occur. Considering the wide discussion in the literature on polyphenols bioavailability, further effort is needed to better define which polyphenols or metabolites reach the colon to assess the prebiotic potential of grape skin polyphenols. In this context, the incorporation of cutting-edge strategies such as encapsulation and in vivo studies will contribute to the improvement of the bioavailability of polyphenols from natural sources and to the knowledge for the development of effective nutraceuticals.

## Figures and Tables

**Table 1:** Polyphenolic compounds identified and quantified in the dried grape skin extract (GSE) by HPLC-DAD.

Compound	Polyphenol Family	Concentration (µg/g)
Delphinidin-3-*O*-glucoside (D3G)	Anthocyanin	212 ± 15
Petunidin-3-*O*-glucoside (Pet3G)	Anthocyanin	196 ± 15
Malvidin-3-*O*-glucoside (M3G)	Anthocyanin	548 ± 35
Quercetin-3-glucoside (Q3G)	Flavonol	28 ± 3
Kaempferol-3-galactoside (K3Gal)	Flavonol	58 ± 5
Kaempferol-3-glucoside (K3G)	Flavonol	254 ± 26
Gallic acid (GA)	Hydroxybenzoic acid	38 ± 4
Syringic acid (SA)	Hydroxybenzoic acid	59 ± 6
Caftaric acid (CA)	Hydroxycinnamic acid	88 ± 8

The values shown are the mean (*n* = 3) ± standard deviation (SD).

**Table 2:** TEER values before and after the bioavailability experiments with GSE solutions through the Caco-2 monolayer.

Experiment	Solution	GSE (mg/mL)	TEER (Ω cm^2^)	
			Before	After
AB	S1	15	602 ± 8	536 ± 31
AB	S2	22	350 ± 25	300 ± 15
BA	S1	15	357 ± 32	340 ± 17
BA	S2	22	320 ± 9	300 ± 9

AB: apical to basolateral experiment; BA: basolateral to apical experiment; GSE: grape skin extract. The values shown are the mean (*n* = 3) ± standard deviation (SD).

**Table 3:** D3G, Pet3G, Q3G, K3Gal and SA concentrations at the apical and basolateral side of the Caco-2 cell monolayer through time in apical to basolateral (AB) experiments.

AB Experiments						
**GSE** **Concentration**	**Polyphenol**	**Apical Side (mg/L)**				
		**0 min**	**75 min**	**90 min**	**115 min**	**180 min**
S1 (15 mg/mL)	D3G	2.1 ± 0.2 ^a^	1.9 ± 0.2 ^a,b^	1.8 ± 0.1 ^b,c^	1.6 ± 0.1 ^c^	1.5 ± 0.1 ^c^
	Pet3G	1.8 ± 0.1 ^a^	1.4 ± 0.1 ^b^	1.2 ± 0.1 ^c^	1.02 ± 0.10 ^c,d^	0.9 ± 0.1 ^d^
	Q3G	0.23 ± 0.01 ^a^	0.18 ± 0.01 ^b^	0.14 ± 0.01 ^c^	0.10 ± 0.00 ^d^	0.08 ± 0.00 ^c^
	K3Gal	0.63 ± 0.02 ^a^	0.52 ± 0.02 ^b^	0.45 ± 0.02 ^c^	0.39 ± 0.01 ^d^	0.35 ± 0.01 ^c^
	SA	0.70 ± 0.03 ^a^	0.54 ± 0.03 ^b^	0.42 ± 0.03 ^c^	0.32 ± 0.03 ^d^	0.23 ± 0.03 ^c^
S2 (22 mg/mL)	D3G	4.7 ± 0.1 ^a^	4.4 ± 0.1 ^b^	4.3 ± 0.1 ^c^	4.05 ± 0.09 ^c^	3.94 ± 0.08 ^c^
	Pet3G	4.4 ± 0.2 ^a^	4.0 ± 0.1 ^b^	3.7 ± 0.1 ^c^	3.33 ± 0.09 ^c^	3.16 ± 0.08 ^c^
	Q3G	0.61 ± 0.01 ^a^	0.52 ± 0.01 ^b^	0.47 ± 0.01 ^c^	0.41 ± 0.01 ^d^	0.37 ± 0.01 ^c^
	K3Gal	1.23 ± 0.02 ^a^	1.09 ± 0.02 ^b^	1.01 ± 0.02 ^c^	0.88 ± 0.02 ^d^	0.79 ± 0.02 ^c^
	SA	1.48 ± 0.06 ^a^	1.21 ± 0.04 ^b^	1.03 ± 0.03 ^c^	0.82 ± 0.02 ^d^	0.67 ± 0.03 ^c^
**GSE** **Concentration**	**Polyphenol**	**Basolateral Side (mg/L)**				
		**0 min**	**75 min**	**90 min**	**115 min**	**180 min**
S1 (15 mg/mL)	D3G	nd	nd	nd	nd	nd
	Pet3G	nd	nd	nd	nd	nd
	Q3G	nd	nd	nd	nd	nd
	K3Gal	nd	nd	nd	nd	nd
	SA	nd	nd	nd	nd	nd
S2 (22 mg/mL)	D3G	nd	nd	nd	nd	nd
	Pet3G	nd	nd	nd	nd	nd
	Q3G	nd	nd	nd	nd	nd
	K3Gal	nd	nd	nd	nd	nd
	SA	nd	nd	nd	nd	nd

D3G: delphinidin-3-*O*-glucoside; Pet3G: petunidin-3-*O*-glucoside; Q3G: quercetin-3-glucoside; K3Gal: kaempferol-3-galactoside; SA: syringic acid. nd, not detected. Each value represents the mean (*n* = 3) ± standard deviation. Lower letters indicate significant differences within the same row (*p* < 0.05).

**Table 4:** D3G, Pet3G, K3Gal and SA concentrations at the basolateral and apical side of the Caco-2 cell monolayer through time in basolateral to apical (BA) experiments.

BA Experiments						
**GSE** **Concentration**	**Polyphenol**	**Basolateral Side (mg/L)**				
		**0 min**	**75 min**	**90 min**	**115 min**	**180 min**
S1 (15 mg/mL)	D3G	2.7 ± 0.2 ^a^	2.6 ± 0.2 ^a,b^	2.5 ± 0.2 ^b,c^	2.4 ± 0.2 ^b,c^	2.3 ± 0.2 ^c^
	Pet3G	4.3 ± 0.1 ^a^	4.0 ± 0.1 ^b^	3.8 ± 0.1 ^b,c^	3.65 ± 0.09 ^c,d^	3.49 ± 0.08 ^d^
	K3Gal	0.26 ± 0.01 ^a^	0.19 ± 0.01 ^b^	0.13 ± 0.01 ^c^	0.08 ± 0.01 ^d^	0.05 ± 0.01 ^c^
	SA	0.84 ± 0.08 ^a^	0.72 ± 0.07 ^a,b^	0.63 ± 0.07 ^b,c^	0.53 ± 0.06 ^c,d^	0.44 ± 0.06 ^d^
S2 (22 mg/mL)	D3G	3.6 ± 0.2 ^a^	3.5 ± 0.2 ^a^	3.4 ± 0.2 ^a^	3.4 ± 0.2 ^d^	3.4 ± 0.2 ^a^
	Pet3G	3.1 ± 0.2 ^a^	3.0 ± 0.2 ^a^	2.9 ± 0.2 ^a^	2.7 ± 0.2 ^a^	2.7 ± 0.2 ^a^
	K3Gal	0.79 ± 0.02 ^a^	0.75 ± 0.02 ^b^	0.71 ± 0.02 ^c^	0.67 ± 0.02 ^d^	0.64 ± 0.01 ^c^
	SA	1.1 ± 0.1 ^a^	1.10 ± 0.09 ^a,b^	1.03 ± 0.09 ^a,b,c^	0.96 ± 0.08 ^b,c^	0.901 ± 0.08 ^c^
**GSE** **Concentration**	**Polyphenol**	**Apical Side (mg/L)**				
		**0 min**	**75 min**	**90 min**	**115 min**	**180 min**
S1 (15 mg/mL)	D3G	nd	nd	nd	nd	nd
	Pet3G	nd	nd	nd	nd	nd
	K3Gal	nd	nd	nd	nd	nd
	SA	nd	nd	nd	nd	nd
S2 (22 mg/mL)	D3G	nd	nd	nd	nd	nd
	Pet3G	nd	nd	nd	nd	nd
	K3Gal	nd	nd	nd	nd	nd
	SA	nd	nd	nd	nd	nd

D3G: delphinidin-3-*O*-glucoside; Pet3G: petunidin-3-*O*-glucoside; K3Gal: kaempferol-3-galactoside; SA: syringic acid. nd, not detected. Each value represents the mean (*n* = 3) ± standard deviation. Lower letters indicate significant differences within the same row (*p* < 0.05).

**Table 5:** M3G, K3G, GA and CA concentrations at the apical and basolateral side of the Caco-2 cell monolayer through time in apical to basolateral (AB) experiments.

AB Experiments							
**GSE** **Concentration**	**Polyphenol**	**Apical Side (mg/L)**					
		**0 min**	**75 min**	**90 min**	**115 min**	**180 min**	
S1 (15 mg/mL)	M3G	6.1 ± 0.3 ^a^	4.5 ± 0.2 ^b^	3.2 ± 0.1 ^c^	2.26 ± 0.06 ^d^	1.53 ± 0.04 ^c^	
	K3G	7.7 ± 0.3 ^a^	7.1 ± 0.2 ^b^	6.7 ± 0.2 ^b,c^	6.4 ± 0.2 ^c^	5.1 ± 0.2 ^d^	
	GA	6.1 ± 0.5 ^a^	5.0 ± 0.5 ^b^	4.1 ± 0.4 ^c^	3.4 ± 0.4 ^c,d^	2.9 ± 0.3 ^d^	
	CA	1.04 ± 0.08 ^a^	0.84 ± 0.07 ^b^	0.67 ± 0.07 ^c^	0.54 ± 0.07 ^d^	0.43 ± 0.07 ^d^	
S2 (22 mg/mL)	M3G	14 ± 1 ^a^	11.6 ± 0.9 ^b^	9.2 ± 0.9 ^c^	7.2 ± 0.9 ^d^	6.0 ± 0.8 ^d^	
	K3G	12.7 ± 0.3 ^a^	11.8 ± 0.3 ^b^	11.3 ± 0.3 ^b,c^	10.6 ± 0.23 ^c^	9.0 ± 0.2 ^d^	
	GA	5.9 ± 0.3 ^a^	5.0 ± 0.3 ^b^	4.4 ± 0.3 ^c^	3.7 ± 0.3 ^d^	3.2 ± 0.3 ^d^	
	CA	1.82 ± 0.03 ^a^	1.54 ± 0.03 ^b^	1.37 ± 0.02 ^c^	1.16 ± 0.02 ^d^	1.01 ± 0.01 ^c^	
**GSE** **Concentration**	**Polyphenol**	**Basolateral Side (mg/L)**					**TE (%)**
		**0 min**	**75 min**	**90 min**	**115 min**	**180 min**	
S1 (15 mg/mL)	M3G	nd	nd	nd	nd	nd	
	K3G	nd	6.2 ± 0.1 ^b^	6.7 ± 0.1 ^c^	7.1 ± 0.1 ^c^	7.4 ± 0.1 ^c^	97 ± 2
	GA	nd	8.1 ± 0.9 ^b^	8.6 ± 0.9 ^b^	9.1 ± 0.9 ^b^	9.4 ± 0.9 ^b^	156 ± 26
	CA	nd	0.06 ± 0.01 ^b^	0.06 ± 0.01 ^b,c^	0.06 ± 0.01 ^b,c^	0.07 ± 0.01 ^c^	7 ± 1
S2 (22 mg/mL)	M3G	nd	0.12 ± 0.01 ^b^	0.13 ± 0.01 ^c,d^	0.14 ± 0.01 ^d,c^	0.15 ± 0.01 ^c^	1.08 ± 0.01
	K3G	nd	6.4 ± 0.1 ^b^	6.8 ± 0.2 ^c^	7.2 ± 0.2 ^d,c^	7.6 ± 0.2 ^c^	60.2 ± 0.9
	GA	nd	8.1 ± 0.9 ^b^	8.6 ± 0.9 ^b^	9.1 ± 0.9 ^b^	9.6 ± 0.9 ^b^	164 ± 23
	CA	nd	0.07 ± 0.01 ^b^	0.07 ± 0.01 ^b,c^	0.08 ± 0.01 ^b^	0.08 ± 0.01 ^b^	4.4 ± 0.4

M3G: Malvidin-3-*O*-glucoside; K3G: kaempferol-3-glucoside; GA: gallic acid; CA: caftaric acid. nd, not detected. Each value represents the mean (*n* = 3) ± standard deviation. Lower letters indicate significant differences within the same row (*p* < 0.05).

**Table 6:** M3G, K3G, Q3G, GA and CA concentrations at the basolateral and apical side of the Caco-2 cell monolayer through time in basolateral to apical (BA) experiments.

BA Experiments							
**GSE** **Concentration**	**Polyphenol**	**Basolateral Side (mg/L)**					
		**0 min**	**75 min**	**90 min**	**115 min**	**180 min**	
S1 (15 mg/mL)	M3G	10.4 ± 0.7 ^a^	9.7 ± 0.6 ^b^	9.1 ± 0.6 ^c^	8.5 ± 0.6 ^d^	8.0 ± 0.6 ^c^	
	K3G	7.6 ± 0.2 ^a^	7.2 ± 0.2 ^b^	6.9 ± 0.2 ^c^	6.6 ± 0.1 ^d^	6.1 ± 0.1 ^c^	
	Q3G	0.28 ± 0.02 ^a^	0.24 ± 0.02 ^b^	0.21 ± 0.02 ^c^	0.18 ± 0.01 ^d^	0.15 ± 0.01 ^c^	
	GA	6.9 ± 0.3 ^a^	6.5 ± 0.3 ^b^	6.2 ± 0.3 ^c^	5.9 ± 0.3 ^d^	5.5 ± 0.2 ^c^	
	CA	1.04 ± 0.03 ^a^	0.91 ± 0.02 ^b^	0.80 ± 0.02 ^c^	0.70 ± 0.02 ^c,d^	0.59 ± 0.02 ^c^	
S2 (22 mg/mL)	M3G	17.5 ± 0.4 ^a^	16.4 ± 0.4 ^b^	15.4 ± 0.3 ^c^	14.5 ± 0.3 ^c,d^	13.7 ± 0.2 ^c^	
	K3G	9.8 ± 0.2 ^a^	9.5 ± 0.2 ^b^	9.3 ± 0.2 ^c^	9.1 ± 0.2 ^d^	8.5 ± 0.2 ^c^	
	Q3G	0.41 ± 0.03 ^a^	0.38 ± 0.03 ^a,b^	0.36 ± 0.03 ^a,b,c^	0.34 ± 0.03 ^b,c^	0.32 ± 0.03 ^c^	
	GA	6.1 ± 0.4 ^a^	5.7 ± 0.4 ^b^	5.4 ± 0.4 ^c^	5.0 ± 0.4 ^d^	4.7 ± 0.3 ^d^	
	CA	1.4 ± 0.1 ^a^	1.3 ± 0.1 ^b^	1.23 ± 0.09 ^c^	1.15 ± 0.08 ^d^	1.08 ± 0.08 ^c^	
**GSE** **Concentration**	**Polyphenol**	**Apical Side (mg/L)**					**TE (%)**
		**0 min**	**75 min**	**90 min**	**115 min**	**180 min**	
S1 (15 mg/mL)	M3G	nd	0.19 ± 0.02 ^a,b^	0.23 ± 0.02 ^b,c^	0.28 ± 0.02 ^c^	0.33 ± 0.02 ^c^	3.2 ± 0.3
	K3G	nd	6.1 ± 0.3 ^b^	7.4 ± 0.3 ^c^	8.7 ± 0.4 ^d^	9.9 ± 0.4 ^c^	130 ± 3
	Q3G	nd	0.009 ± 0.001 ^b^	0.010 ± 0.001 ^b,c^	0.011 ± 0.001 ^c^	0.011 ± 0.001 ^c^	4.1 ± 0.1
	GA	nd	8.1 ± 0.3 ^a,b^	9.4 ± 0.2 ^b,c^	10.5 ± 0.3 ^c,d^	11.3 ± 0.3 ^d^	164 ± 6
	CA	nd	0.08 ± 0.01 ^b^	0.09 ± 0.01 ^c^	0.10 ± 0.01 ^d^	0.12 ± 0.01 ^c^	11.2 ± 0.8
S2 (22 mg/mL)	M3G	nd	0.18 ± 0.01 ^b^	0.22 ± 0.01 ^c^	0.26 ± 0.01 ^d^	0.30 ± 0.01 ^c^	1.73 ± 0.04
	K3G	nd	6.8 ± 0.2 ^a,b^	8.1 ± 0.2 ^b,c^	9.4 ± 0.3 ^c,d^	10.6 ± 0.3 ^c^	109.0 ± 0.9
	Q3G	nd	0.013 ± 0.001 ^b^	0.013 ± 0.001 ^b,c^	0.014 ± 0.001 ^c,d^	0.015 ± 0.001 ^d^	3.6 ± 0.1
	GA	nd	8.3 ± 0.5 ^a,b^	9.6 ± 0.5 ^b,c^	10.7 ± 0.6 ^c^	11.5 ± 0.7 ^c^	188 ± 3
	CA	nd	0.08 ± 0.01 ^a,b^	0.10 ± 0.01 ^a,b,c^	0.11 ± 0.01 ^b,c^	0.12 ± 0.01 ^c^	8.7 ± 0.9

M3G: Malvidin-3-*O*-glucoside; K3G: kaempferol-3-glucoside; Q·G: quercetin-3-glucoside; GA: gallic acid; CA: caftaric acid. nd, not detected. Each value represents the mean (*n* = 3) ± standard deviation. Lower letters indicate significant differences within the same row (*p* < 0.05).

**Table 7:** Apparent permeability (P_app_) coefficients in the apical to basolateral (AB) and basolateral to apical (BA) experiments and the efflux ratio (ER) of M3G, K3G, GA and CA in the Caco-2 model at S1 (15 mg/mL) and S2 (22 mg/mL) GSE concentrations.

Polyphenol	P_app AB_ (10^−4^ cm/s)		P_app BA_ (10^−4^ cm/s)		ER	
	S1	S2	S1	S2	S1	S2
M3G	nd	0.15 ± 0.01	0.33 ± 0.02	0.18 ± 0.01	nd	1.2
K3G	11.6 ± 0.2	7.3 ± 0.4	12.3 ± 0.3	9.7 ± 0.3	1.1	1.3
GA	16.9 ± 0.7	18 ± 2	11.5 ± 0.6	12.90 ± 0.05	0.7	0.7
CA	0.74 ± 0.03	0.51 ± 0.02	0.93 ± 0.07	0.73 ± 0.08	1.3	1.4

M3G: Malvidin-3-*O*-glucoside; K3G: kaempferol-3-glucoside; Q·G: quercetin-3-glucoside; GA: gallic acid; CA: caftaric acid. nd, not detected. Each value represents the mean (*n* = 3) ± standard deviation.

## Data Availability

The original contributions presented in this study are included in the article. Further inquiries can be directed to the corresponding author.

## Abbreviations

The following abbreviations are used in this manuscript:

A	Surface area of the Caco-2 cell monolayer
AB	Apical to basolateral side
AP	Apical side
BA	Basolateral to apical side
BL	Basolateral side
C	Polyphenol concentration at the acceptor side
C_0_	Polyphenol initial concentration at the donor side
CA	Caftaric acid
Caco-2	Caucasian colon adenocarcinoma cell line
D3G	Delphinidin-3-*O*-glucoside
DAD	Diode array detector
DMEM	Dulbecco’s modified eagle’s medium
DMSO	Dimethyl sulfoxide
dQ	Change of polyphenol concentration at the acceptor side
dt	Variation in time
ER	Efflux ratio
FBS	Fetal bovine serum
GA	Gallic acid
GSE	Grape skin polyphenolic extract
HBSS	Hank’s balanced salt solution
HEPES	4-(2-Hydroxyethyl)-1-piperazineethanesulfonic acid
HPLC	High performance liquid chromatography
K3G	Kaempferol-3-glucoside
K3Gal	Kaempferol-3-galactoside
LSD	Fisher‘s Least Significant Difference
M3G	Malvidin-3-*O*-glucoside
MIN	Minute
MTT	3-[4,5-Dimethylthiazol-2-yl]-2,5 diphenyl tetrazolium bromide
NEAA	Non-essential amino acid
P_app_	Apparent permeability coefficient
PBS	Phosphate-buffered saline solution
Pet3G	Petunidin-3-*O*-glucoside
Q3G	Quercetin-3-glucoside
S1	Solution 1 (15 mg grape skin polyphenolic extract/mL)
S2	Solution 2 (22 mg grape skin polyphenolic extract/mL)
SA	Syringic acid
SD	Standard deviation
TE	Transport efficiency
TEER	Transepithelial electrical resistance
V_R_	Volume of the solution of the acceptor side
